# Fc glycan sialylation of biotherapeutic monoclonal antibodies has limited impact on antibody‐dependent cellular cytotoxicity

**DOI:** 10.1002/2211-5463.13267

**Published:** 2021-10-19

**Authors:** Elaine Branstetter, Robert J. Duff, Scott Kuhns, Rupa Padaki

**Affiliations:** ^1^ Department of Attribute Sciences Amgen Inc. Thousand Oaks CA USA

**Keywords:** glycan profiling, antibody‐dependent cellular cytotoxicity, sialylation, IgG glycosylation, monoclonal antibody, mAb

## Abstract

It has been well documented that the terminal sugars of Fc glycans can play a critical role in the safety and efficacy of therapeutic mAbs. However, many of the effects of highly heterogeneous Fc glycan structures have yet to be fully characterized. Different glycosylation patterns can affect Fc‐dependent activities, such as the ability of mAbs to bind Fcγ receptors on the effector cell surface, which is critical to immune effector functions, such as antibody‐dependent cellular cytotoxicity (ADCC). Previous studies on the impact of sialic acid in the Fc glycan on ADCC have not resulted in consistent conclusions. In our study, we tested sialic acid‐enriched species from a chimeric murine/human kappa light chain IgG1 (mAb1) with known Fcγ receptor IIIa binding and ADCC activities. These enriched species contained up to a fourfold increase in sialic acid‐containing glycans relative to the typical levels present in therapeutic mAbs, along with other attributes such as oxidized and deamidated species. The ADCC analysis of sialylated and asialo mAb1 provided herein shows evidence that sialic acids have little or no impact on ADCC activity. Altogether, our results highlight the value of novel glycan engineering strategies in designing therapeutic mAbs with high‐quality attributes and in improving production process controls.

## Graphical abstract

In this study, it was hypothesized that ADCC activity for mAb1 has little or no dependence on the sialylation level of the Fc glycan. Experiments using the acidic fractions of mAb1 from cation exchange chromatography and sialyltransferase treatments, both enriching for sialic acid‐containing species, indicate no impact of sialic acid content on ADCC activity.

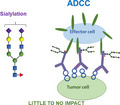

**Abbreviations**
2‐AA: 2‐aminobenzoic acid
ADCC: antibody‐dependent cellular cytotoxicity
CEX: cation exchange
CHO: Chinese hamster ovary
FcγRIIIa (158V)–GST: FcγRIIIa (158V)‐glutathione‐S‐transferase
HILIC: hydrophilic interaction liquid chromatography
UPLC: ultra‐performance liquid chromatography

Glycosylation is a critical quality attribute of therapeutic proteins and is an important posttranslational modification. For mAbs, complex, highly branched glycans are attached to a conserved asparagine residue on a (mAb’s) Fc region. The Fc portion of IgG mAbs is known to interact with distinct Fcγ receptors (FcγRs), which in turn impact the immunological activities of the molecule, such as antibody‐dependent cellular cytotoxicity (ADCC). Glycans attached to the Fc portion play a key role in this interaction. ADCC activity correlates well with the varying levels of complex/hybrid afucosylated and high‐mannose‐type glycans [1]; however, there are limited data for the potential impact of sialic acids on ADCC.

Terminal sialic acid glycans have two forms, *N*‐glycolylneuraminic acids or *N*‐acetylneuraminic acids, and are nine‐carbon carboxylic acids that are found mostly in the terminal nonreducing end of asparagine‐linked glycan chains on the surfaces of molecules and cells [2]. Synthesis of sialic acids begins in the cytosol, and the intact monosaccharides are transferred to the terminal β‐galactoses of nascent oligosaccharides catalyzed by sialyltransferases in the Golgi. The sialic acid and the β‐galactose may be linked by either an α2,3 or α2,6 linkage dependent upon what type of sialyltransferase catalyzes the synthesis [3]. Chinese hamster ovary (CHO) cells contain only α2,3 sialyltransferases; therefore, sialylated glycans derived from CHO cell lines contain only α2,3‐linked sialic acids [4]. Because the human IgG1 mAb used in this study was expressed in a CHO cell line, all sialylated molecules have sialic acids linked in an α2,3 orientation to the β‐galactose residue at the nonreducing terminus of the glycan chain.

Scallon *et al*. [5] observed that sialylation of Fc glycans for IgG1 kappa does in fact negatively influence ADCC activity. Key mAbs’ Fc glycans, most notably afucosylated and high‐mannose species, are vital to ADCC activity [4]. Recent literature reports indicate that increased terminal sialylation may lead to decreased ADCC activity and may affect target binding [5, 6, 7], whereas other studies contradict these findings and suggest that sialylation levels show no impact on ADCC activity [8, 9]. Thomann *et al*. [8] observed no impact on either ADCC or FcγRIIIa binding on samples with a 47% difference in sialic acid levels. Another study performed by Boyd *et al*. [9] also revealed sialylation had no influence on ADCC or FcγRIIIa binding, although the sialic level variation was significantly less than 47%. In yet another study, Li *et al*. [10] observed that sialylation adversely impacts ADCC in the presence of core fucosylation, but not in afucosylated glycans.

In this study, it was hypothesized that ADCC activity for a core‐fucosylated therapeutic antibody candidate, mAb1, has little or no dependence on the α2,3‐linked sialic acid content of the Fc glycan. This antibody was chosen for this study in part because it was found to vary in sialic acid content through the normal production process and therefore had relevance to address the specific impact on ADCC activity. Attribute‐focused experiments using the acidic fractions of mAb1 as collected from cation exchange (CEX) chromatography, together with enzymatic treatments that aid in selectively enriching for sialic acid‐containing species, were designed to assess the impact of sialic acid content on ADCC. The results of this study suggest process development efforts that may be given consideration to control for the most relevant product quality attributes for the desired biological activities.

## Materials and methods

### Materials

A human IgG1 (mAb1) expressed in CHO cells was produced at Amgen Inc. (Thousand Oaks, CA, USA) and used for the glycan analysis. Reagent grade 2‐aminobenzoic acid (2‐AA), anthranilic acid‐(phenyl‐^13^C_6_), 2‐picoline borane and ammonium hydroxide were obtained from Sigma‐Aldrich (Milwaukee, WI, USA). Boric acid was obtained from Alfa Aesar (Ward Hill, MA, USA), and sodium acetate trihydrate from JT Baker (Center Valley, PA, USA). Sodium cyanoborohydride reagent and HPLC grade water, acetonitrile and formic acid and were purchased from Fisher Scientific (Pittsburgh, PA, USA).

All chemicals throughout the article are reagent grade unless otherwise specified.

### Fraction collection from CEX chromatography

Charged variants of mAb1 were separated on a CEX column (054993; Thermo Fisher Scientific, Waltham, MA, USA) with elution by a salt gradient. Solvent A consisted of 20 mm sodium phosphate (pH 7.2) prepared with 1 m sodium phosphate, monobasic (S0241; Teknova, Hollister, CA, USA) and 0.5 m sodium phosphate, dibasic (S02119; Teknova) filtered through a 0.2‐μm filter (430517; Corning, Corning, NY, USA). Solvent B consisted of 20 mm sodium phosphate, 500 mm sodium chloride (pH 7.2) prepared with 5 m sodium chloride solution (S0250; Teknova) filtered through a 0.2‐μm filter. The column temperature was maintained at 30°C, and the detection was set at 280 nm. The flow rate was 1.0 mL·min^−1^ with an initial 96.5% solvent A held for 2 min lowered to 84% solvent A over 10 min, and a 2‐min wash at 5% solvent A before a 5‐min reequilibration. Acidic, main and basic peaks were collected and reinjected onto the system to check for purity, and individual peak fractions were then subjected to N‐glycan and peptide mapping and ADCC analysis.

### Sialidase digestion for release of sialic acids

**Fig. 1:**
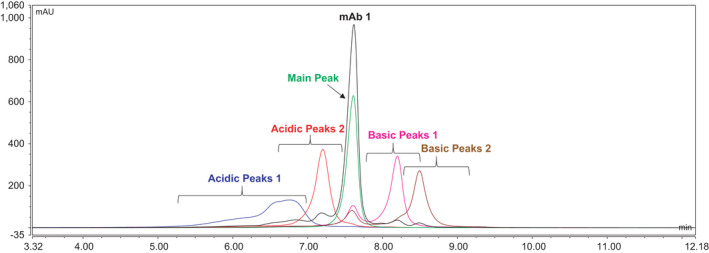
Chromatographic overlay showing profiles of unfractionated mAb1 and reinjected peaks of previously collected fractions of main peak and acidic and basic peaks 1 and 2 after separation by CEX chromatography.

The acidic peak fractions 1 and 2 from the CEX chromatography separation (Fig. 1) were incubated with sialidase (GK80040; Prozyme, Hayward, CA, USA) at 37°C for 1 h. For each sample, an aliquot containing 750 µg was mixed with 20 µL 5 U·mL^−1^ sialidase, 40 µL of 5× sodium phosphate buffer (WS0049; Prozyme) and the appropriate amount of water to bring the total volume to 200 µL. Controls were prepared by substituting water for sialidase. All samples and controls were then buffer exchanged into 154 mm sodium chloride, 25 mm sodium citrate dehydrate, 0.07% (w/v) polysorbate 80 (pH 6.5) using a 100 MWCO centrifugal filter (UFC510024; EMD Millipore, Burlington, MA, USA). A 100‐µg aliquot of each sample and control was run on a hydrophilic interaction liquid chromatography (HILIC) N‐glycan mapping method to measure the % sialylation (see later description of method).

### Release of N‐glycans from IgG1

The buffer‐exchanged, desialylated peak fraction samples and controls described earlier were incubated with PNGase F for 2 h at 37 °C to release N‐glycans. For each sample, an aliquot containing 100 μg of IgG1 was mixed with a solution containing 2 μL of 10 U·mL^−1^ recombinant PNGase F (GKE‐5010B; Prozyme), 6 μL of 5× sodium phosphate buffer (WS0010; Prozyme) and the appropriate amount of water to bring the total volume to 30 μL. The reaction yielded approximately 1.3 nmol N‐glycans prepared for derivatization.

### Derivatization of N‐glycans

The released N‐linked glycans described earlier were treated separately with an aliquot of a solution containing 20 mg·mL^−1^ 2‐AA, 200 mm sodium cyanoborohydride (8.18053.0010; Fisher Scientific) and incubated at 80 °C for 75 min. The 2‐AA reagent was prepared by dissolving 1.0 g of 2‐AA (10680; Sigma‐Aldrich) and 0.63 g of sodium cyanoborohydride in 20 mL 4% (w/v) sodium acetate, 2% (w/v) boric acid in a methanolic solution. An additional 30 mL of methanol was added to the solution after being dissolved to make the final 2‐AA labeling solution. Specifically, an aliquot (50 μL) of 2‐AA reagent was added to a 30‐μL aliquot of a solution containing the IgG1 glycans derived from a PNGase F deglycosylation reaction. The 2‐AA derivatization reaction proceeded for 75 min at 80 °C.

### HILIC

The 2‐AA‐derivatized mAb1 samples and controls described earlier were profiled with a Waters Acquity Ultra‐Performance Liquid Chromatography (UPLC) H class System (Milford, MA, USA) equipped with a Waters BEH Glycan column (1.7 µm, 2.1 × 150 mm) (Milford, MA, USA). Solvent A consisted of 100 mm ammonium formate prepared with formic acid (1116701000; Fisher Scientific) and brought to pH 3.0 with ammonium hydroxide (338818; Sigma). Solvent B consisted of 100% acetonitrile (MX0486‐6; Fisher Scientific). The column temperature was maintained at 35°C, and the fluorescence detection was set at *λ*_ex_ = 360 nm and *λ*_em_ = 425 nm. The flow rate was 0.25 mL·min^−1^ with an initial 22% solvent A ramped up to 40% solvent A in 111 min, followed by a 6.5‐min wash at 90% solvent A, then a 5‐min stepdown to 22% solvent A and a 25‐min final reequilibration. The flow rate was decreased by 20% for the wash step to avoid approaching the pressure limit of the system.

### ADCC analysis

ADCC activity was determined using WIL2‐S lymphoma cells expressing high levels of CD20 as target cells and NK92‐M1 cells, stably transfected with the high‐affinity human CD16 allele (158V), as effector cells. Target cells were loaded with calcein‐AM (C1359; Sigma), which readily enters the cells, where it is cleaved by intracellular esterase to the polar, lipid‐insoluble fluorescent product, calcein, which is retained by cells with intact membranes. When the calcein‐loaded target cells die through ADCC, the calcein is released into the media. The amount of supernatant fluorescence detected in a plate reader is proportional to the amount of target cell death. The % relative cytotoxicity of test samples was determined by comparing the half maximal effective concentration (EC_50_) of test sample curves with the reference standard curve. The reference standard and test samples were serially diluted over eight concentration levels in RPMI 1640 assay medium with low IgG FBS to the range of 0.016–100 ng·mL^−1^ of the final plate well concentration to generate a dose–response curve. Effector NK92‐M1 and target (WIL‐2S) cells were prepared in a combined cell suspension at an effector/target cell ratio of 25:1. The plate was then incubated in a humidified incubator at 5% CO_2_ and 37 °C for 1.0 h. At the end of the incubation, the plate was centrifuged for 5 min to collect the supernatant. Fluorescence released into the media was detected by an EnVision (Perkin Elmer) plate reader. Data were fitted to the mean emission values using a four‐parameter curve fit using SoftMax Pro 7 and reported as percent relative activity (EC_50_ standard/EC_50_ sample) per common bioassay practices. Each sample was tested in three independent assays, and the sample result was reported as the mean of the three determinations. This method has been described in an earlier paper (see Kuhns *et al*. [11]).

### FcγRIIIa binding analysis

The FcγRIIIa (158V) binding assay is a bead‐based amplified luminescent proximity homogeneous assay (AlphaLISA; Perkin Elmer) that detects bimolecular interactions. The assay contains two bead types, an acceptor bead and a donor bead. The acceptor beads contain the fluorophore europium chelate and are coated with a hydrogel that contains glutathione, which binds recombinant human FcγRIIIa (158V)‐glutathione‐*S*‐transferase [FcγRIIIa (158V)–GST]. The donor beads are coated with a hydrogel that contains phthalocyanine, a photosensitizer and streptavidin, which binds to biotinylated human IgG1. When FcγRIIIa (158V)–GST and the biotinylated human IgG1 bind together, they bring the acceptor and donor beads into close proximity. When the acceptor and donor beads are in close proximity, luminescence is produced and measured in a plate reader equipped for luminescence signal detection. When mAb1 was present at sufficient concentrations to inhibit the binding of FcγRIIIa (158V)–GST to the biotinylated human IgG1, a dose‐dependent decrease in light is measured. Data were fitted to the mean emission values using a four‐parameter curve fit using SoftMax Pro 7 and reported as percent relative activity (EC_50_ standard/EC_50_ sample). Each sample was tested in three independent assays, and the sample result was reported as the mean of the three determinations.

### Sialic acid enrichment

A total of 2000 µg of mAb1 was buffer exchanged into 100 mm Tris–HCl (pH 8.0, 1×) reaction buffer using a 100 MWCO centrifugal filter (UFC510024; EMD Millipore). Eight separate reactions of 20 µL of 10 mg·mL^−1^ buffer‐exchanged mAb1, 15 µL 10 mg·mL^−1^ CMP‐*N*‐acetylneuraminic acid, 10 µL 5 mg·mL^−1^ α‐2,3‐sialyltransferase (S1951‐1UN; Sigma) and 55 µL 1× reaction buffer were added to eight 0.5‐mL microcentrifuge tubes and incubated at 37 °C for 30 min. Two controls were prepared, one as the earlier reaction with reaction buffer used in place of enzyme and another by adding 200 µL of 10 mg·mL^−1^ mAb1 to 0.5‐µL microcentrifuge tube. Both controls were incubated at 37 °C for 30 min.

### Peptide mapping method

Tryptic peptides resulting from digested protein were injected and separated on an Acquity UPLC system (Waters Corp., Milford, MA, USA) using Acquity UPLC BEH C4 RP‐column, 1.7 μm, 2.1 × 150 mm (Waters Corp.) at 50°C and analyzed online with an LTQ/Orbitrap Elite mass spectrometer (Thermo Fisher Scientific). The mobile phases consisted of 0.1% formic acid in water (solvent A) and 0.1% formic acid in acetonitrile (solvent B). The chromatography was carried out using a linear gradient from 0.5% to 55% solvent B in 50 min at a flow rate of 400 μL·min^−1^. Data acquisition was controlled by Xcalibur™ software (Thermo Fisher Scientific). This method has been described in an earlier paper (see Duff *et al*. [12]).

## Results and Discussion

The following four sections describe the sample collection and purity analysis, the impact of mAb1 desialylation and hypersialylation on ADCC activity, and nonglycan attribute assessments. The ADCC assay used in this study was purposefully designed to use a high‐expressing target cell line and effector function cells expressing the high‐affinity CD16 allele to maximize the ADCC activity and thereby maximize the sensitivity to varying levels of sialic acid.

### Sample collection and purity analysis

To obtain samples with enriched Fc sialylation, we separated individual charge variants of mAb1 and collected them by CEX chromatography. Purity was assessed through reinjection of an aliquot of the collected fractions. Reinjected acidic, main and basic peaks were highly pure and included little, if any, contamination by neighboring peaks (Fig. 1). The acidic charge variants (acidic peaks 1 and 2) were then analyzed by a HILIC N‐glycan mapping procedure to determine sialic acid content. The data showed that acidic peaks 1 and 2 contained 17.4% and 8.0% sialylation, respectively. The predominant species, contributing 50.7% and 36.0% of the total sialylation for acidic peaks 1 and 2, respectively, were complex biantennary glycans containing core fucose and terminating with two galactose residues.

### Impact of mAb1 desialylation

**Fig. 2:**
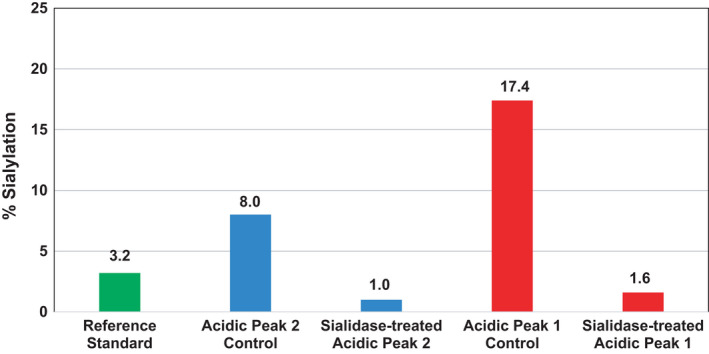
Graph showing % sialic acid of reference standard and acidic peaks 1 (red) and 2 (blue) collected from CEX chromatography with and without treatment with sialidase to cleave sialic acids, *n* = 1. % Sialylation is decreased for sialidase‐treated acidic peaks 1 and 2 from 17.4% and 8.0% to 1.6% and 1.0%, respectively, indicating sialylation is almost absent in sialidase‐treated samples.

To study the impact of sialylation on ADCC, we removed the terminal sialic acids enzymatically. A portion of each of the collected acidic peaks 1 and 2 was then subjected to treatment with sialidase. The sialidase‐treated and untreated acidic peaks 1 and 2 were then analyzed by HILIC N‐glycan map. Sialidase digestion yielded an eightfold decrease in sialylation in acidic peaks 1 and 2 (Fig. 2).

Scallon *et al*. [5] report that analysis of an IgG1 kappa light chain mAb with 26% sialylation requires approximately sixfold higher concentration for the same degree of cell lysis as the same mAb with 0% sialylation. To determine whether this level of sialylation is an impactful attribute to the activity of mAb1, we evaluated aliquots of sialidase‐treated and untreated acidic peaks 1 and 2 using a cell‐based ADCC assay (see Materials and Methods).

**Fig. 3:**
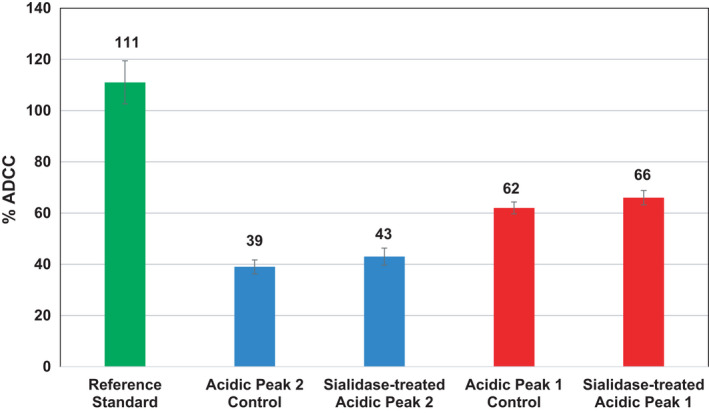
Graph showing the relative ADCC of reference standard and acidic peaks 1 (red) and 2 (blue) with and without sialidase treatment [error bars represent standard deviation (SD), *n* = 3]. The % ADCC is within the SD between the sialylated and desialylated peaks for both acidic peaks 1 and 2, indicating sialylation has little to no impact on ADCC activity.

Acidic peak 1 variants with an eightfold difference in sialylation (17.4% and 1.6%) show relative ADCC activities of 62% and 66%, respectively. Similarly, acidic peak 2 variants with an eightfold difference in sialylation (8.0% and 1.0%) show a relative ADCC activity of 39% and 43%, respectively (Fig. 3). Although it is noted that there is an overall decline in ADCC activity in the acidic fractions relative to the reference sample, that drop is attributable to an impact to antigen binding (see Supporting Information). The central observation from these data was that there was no significant difference in relative ADCC for the variants of acidic peak 1 nor the variants of acidic peak 2 despite the eightfold differences in sialic acid levels for both peak subvariants. If sialylation were critical to the relative ADCC, it would be expected that an eightfold difference would result in a significant difference in ADCC. Because there is no significant difference in relative ADCC observed for variants of acidic peaks 1 and 2, it is concluded that sialylation is not an attribute that impacts the relative ADCC of mAb1.

### Nonglycan attribute assessments

The relative ADCC of acidic peak 1 and 2 variants is significantly lower than that of the mAb1 reference standard at 111%. Acidic peaks of mAb1 are known to contain other attributes such as asparagine deamidation and methionine oxidation in the Fc region that may impact ADCC. Peptide mapping experiments were performed to detect levels of Fc deamidation and oxidation in the reference standard control and the acidic peak 1 and 2 variants. The levels of Fc deamidation in acidic peaks 1 and 2 did not show a significant increase from the control. The level of Fc methionine oxidation, however, increased from 3.5% in the reference standard to 10.2% and 13.1% in acidic peaks 1 and 2, respectively (see Supporting Information). Due to the magnitude of the decline in potency observed, likely oxidation in the Fc region is responsible for the decrease in ADCC activity. Oxidation in the Fc region is known to impact FcγRIIIa binding, thus decreasing ADCC activity. This, along with the drop in antigen binding, contributes to the decrease in relative ADCC in the control and sialidase‐treated acidic peaks 1 and 2 from the reference standard.

The relative ADCC for untreated acidic peak 2 increases slightly from 39% to 43% when treated with sialidase. Untreated acidic peak 1 increases slightly from 62% to 66% when treated with sialidase. Upon sialidase digestion for both acidic peaks, the slight increase in ADCC is within the variability of the assay.

### Hypersialylation of mAb1

**Fig. 4:**
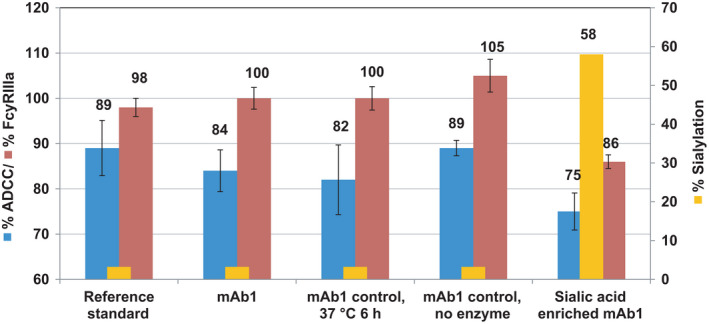
Graph showing % ADCC (blue) and % FcγRIIIa (red) of reference standard, hypersialylated mAb1 and controls (error bars represent standard deviation, *n* = 3). % Sialylation for reference standard, mAb1 and mAb1 controls show % ADCC and % FcγRIIIa values within standard deviation when compared with mAb1 enriched to 58% sialylation. The data indicate that sialylation has little to no impact on ADCC activity or FcγRIIIa binding.

Scallon *et al*. [5] also reported that 41% sialylated material required an approximately sixfold higher concentration than 29% sialylated material to achieve the same level of cell lysis. To better understand the correlation between sialylation and FcγRIIIa binding and ADCC, we used sialyltransferase to increase the sialylation levels of mAb1. HILIC N‐glycan map determined that the levels were increased from ˜3% in the reference standard and controls to 58%, well above the levels achieved with the collected CEX acidic peak fractions (Fig. 4). Reference standard, mAb1, mAb1 controls and sialic acid‐enriched mAb1 were subjected to ADCC and FcγRIIIa binding analysis. The FcγRIIIa analysis shows the reference standard, and controls ranged from 98% to 105% compared with 86% in hypersialylated mAb1. The relative ADCC analysis shows the reference standard and controls ranged from 82% to 89% compared with 75% in hypersialylated mAb1. The level of FcγRIIIa binding of hypersialylated mAb1 falls slightly outside of the assay variability compared with the low end of the range of the reference standard and controls. The ADCC analysis revealed the relative ADCC of the hypersialylated mAb1 was within the assay variability of the low end of the range of the reference standard and controls. These modest decreases could be attributable to other attributes affected as part of the hypersialylated sample that may impact target binding. However, to justify sialylation as being an impactful attribute to either FcγRIIIa binding or ADCC activity, a much larger variance would be expected to be seen in both of these cases with a 20‐fold difference in sialylation. These observations agree with reports by Thomann *et al*. [8, 9] and Boyd *et al*. [8, 9] that sialylation is not impactful to ADCC.

## Conclusions

Contrary to some previous reports [5, 6, 7, 10, 13], this work has provided direct evidence supporting the hypothesis that sialic acids play little or no role in ADCC activity of therapeutic mAb. The modest decline in ADCC activity in the acidic peaks from the ion exchange chromatography enrichment indicate that attributes such as deamidation and/or oxidation rather than sialylation in the acidic peaks are more likely influencing ADCC. Further experiments with the mAb1 material in the hypersialylated form using sialyltransferase indicated no significant impact on FcγRIIIa binding or relative ADCC activity.

This work adds to the body of literature that supports the hypothesis that sialic acid is not impactful to ADCC, but it is also recognized that there is the possibility that sialic acid impact could be dependent on the specific antibody or specific target antigen, which may account for the differing conclusions on this topic in the literature. This antibody was chosen for this study because it was found to vary in sialic acid content through the normal production process and therefore had relevance to address the specific impact on ADCC activity. The results of this study can similarly be used to inform process development efforts to control the most relevant product quality attributes for the deserved biological activities.

## Conflict of interest

The authors declare no conflict of interest.

## Author contributions

RJD designed the project, EB and RP acquired the data, SK and RJD interpreted the data, and EB, RJD, and SK wrote the paper.

## Data Availability

No other data are available.
